# VEGF-c expression in an *in vivo* model of orthotopic endometrial cancer and retroperitoneal lymph node metastasis

**DOI:** 10.1186/1477-7827-11-49

**Published:** 2013-05-21

**Authors:** Yong-Wen Huang, Li-Qun Xu, Rong-Zhen Luo, Xin Huang, Teng Hou, Yan-Na Zhang

**Affiliations:** 1 Department of Gynecology, State Key Laboratory of Oncology in South China, Sun Yat-sen University Cancer Center, Guangzhou, Guangdong 510060, P. R. China; 2 Department of Pathology, State Key Laboratory of Oncology in South China, Sun Yat-sen University Cancer Center, Guangzhou, Guangdong 510060, P. R. China

**Keywords:** Vascular endothelial growth factor c, Endometrial cancer, Fluorescence real-time quantitative PCR, Disease animal model, Animal, Rabbit

## Abstract

**Background:**

Retroperitoneal lymph node (RLN) metastasis is an important indicator of endometrial cancer (EC) prognosis. Because vascular endothelial growth factor c (VEGF-c) is known to influence lymphangiogenesis and thereby lymph node metastasis, this study assessed the relationship of VEGF-c mRNA expression with RLN metastasis in EC.

**Methods:**

The uterine muscularis mucosae of New Zealand white rabbits were inoculated with a VX2 tumor cell suspension after which they were sacrificed at 15, 18, 21, 24, 27 and 30 days. Control groups consisted of those receiving no treatment or an injection of saline. EC and metastatic RLN tissues along with peripheral blood samples were collected, and VEGF-c mRNA expression was evaluated using fluorescence real-time quantitative PCR.

**Results:**

The establishment of an in vivo model of EC with complete RLN metastasis was pathologically confirmed at day 21 post-injection with VX2 cells. As compared to the control groups, VEGF-c mRNA expression increased significantly over time in the tumor site, RLN, and peripheral white blood cells of EC rabbits. Significantly higher VEGF-c mRNA expression was observed in metastatic RLNs as compared to those without metastasis (*P* < 0.001). In addition, increased VEGF-c mRNA expression was observed in peripheral white blood cells of rabbits with RLN metastasis (*P* < 0.002).

**Conclusion:**

Injection of a VX2 cell suspension is a simple method of establishing an in vivo EC model. VEGF-c may play an important role in the development of EC and its metastasis to RLN and may be useful marker to predict RLN metastasis.

## Background

Endometrial cancer (EC) is one of the most common malignancies of the female reproductive system [1]. Whereas patients with early-stage EC have a good prognosis, those with advanced EC usually develop retroperitoneal lymph node (RLN) metastasis with a 5-year survival rate, ranging from 30 to 40% [2-4]. Thus, it is crucial to develop new methods for predicting RLN metastasis in EC that can inform clinicians in selecting treatment modalities.

Analysis of a panel of angiogenic factors, including vascular endothelial growth factors (VEGF-A, -B, -C, D), matrix metalloproteinase-2 (MMP-2), and basic fibroblast growth factor (bFGF), in 16 gynecological cancer cell lines revealed that VEGF-c expression was significantly correlated with cell migration as well as MMP-2 levels [5]. VEGF-c influences endothelial cell growth, migration and survival; its signaling is medicated by the VEGF receptors-2 and -3 (VEGFR-2 and VEGFR-3) on the surface of endothelial cells [6]. VEGF-c also regulates lymphangiogenesis and promotes metastasis to lymph nodes as well as distant organs [6-8]. Because preoperative VEGF-c levels correlated with tumor stage and were an independent risk factor for survival in patients with certain types of EC [9], circulating VEGF-c levels may be useful as a prognostic tool to identify those EC patients most at risk for RLN metastasis. Furthermore, poorer prognosis was associated with VEGF-c expression in esophageal squamous cell carcinoma [10], and its upregulation was also noted in the metastasis of cervical [11] and colorectal [12] cancers.

In the present study, an in vivo model of RLN metastasis of EC was established by injecting a VX2 tumor cell suspension into the myometrium of New Zealand white rabbits. Fluorescence real-time quantitative PCR (RT-PCR) was employed to detect VEGF-c mRNA expression in EC tissues, RLNs, and peripheral white blood cells at various time points after injection. These data may provide a theoretical basis for further studies to evaluate VEGF-c as a marker of RLN metastasis in EC.

## Methods

### Animals

A total of 49 female New Zealand white rabbits weighing 2-2.5 kg were purchased from the Huadong Xinhua Experimental Animal Center in Guangzhou (License No: 0098816). One rabbit was used as the source of VX2 tumor cells while the remaining animals were used to establish the in vivo model. The animals were individually housed, allowed free access to standard laboratory food and water, and subjected to daily 12-hour light and dark cycles. The animal protocol used in this study was approved by the Center’s Animal Welfare Committee of Sun Yat-sen University Cancer Center, Guangzhou, Guangdong, China.

### VX2 cell isolation

VX2 cells were kindly purchased from the Cell Bank of the Sun Yat-sen University. Stocks of VX2 cells were mixed in 5 mL RPMI 1640, resulting in a VX2 solution of approximately 1 × 10^10^ cells/mL, 0.2 mL of the cell suspension was injected into the quadriceps femoris of one rabbit. After 21 days, a solid mass was removed from the injection site, washed in normal saline, and placed in RPMI 1640. Areas with active growth were selected, and the tissue was cut into pieces of 0.5-1 mm in diameter. After vortexing, the solution (1 × 10^10^ cells/mL) was transferred to a syringe with a lumbar puncture needle.

### Establishment of an animal model of EC with RLN metastasis

Animals in the experimental group were anesthetized with 3% pentobarbital sodium at 1 mL/kg via the ear vein and then placed in a supine position. After sterilization, a mid-line incision was made on the lower abdomen. After the uterus was exposed, 0.5 mL of the VX2 cell solution (1 × 10^10^ cells/mL) was injected into the muscularis mucosae of the myometrium 1 cm away from the cervix. The injection site was sutured, and the wound was closed with a 1-0 suture.

Six animals in the normal control group were randomly selected; they did not receive anesthesia or surgery. For the saline group, six rabbits received an injection of 0.5 mL of normal saline into the muscularis mucosae of the myometrium in place of the VX2 cell solution.

### Sample collection and pathological examination

At 15, 18, 21, 24, 27 and 30 days post-injection of the VX2 cell solution, rabbits were sacrificed by aeroembolism (injection of air into the ear vein) (n = 6 per time point). At 30 days, animals in the normal control and saline groups were sacrificed by the same method. EC and RLN tissues were observed macroscopically, and the long diameter (a) and short diameter (b) were measured to calculate EC and RLN volume using the following equation: V = a × b^2^/2. Under aseptic conditions, the uterus, EC, and RLNs were collected. Half of the tissue was placed in Trizol (Invitrogen, CA, USA) and stored in liquid nitrogen and then at -80°C, and the other half was fixed in 10% formalin and embedded in paraffin. Sections were obtained and stained with hematoxylin and eosin (H&E). The stained EC and metastatic RLN tissues were independently observed under a light microscope by two pathologists, who were blinded to the treatment conditions.

At the time of sacrifice, a blood sample was also collected, incubated with a blood cell separation solution, Histopaque-1107 (Sigma Aldrich, St. Louis, MO, USA), and centrifuged at 3500 r/min for 10 min. The white blood cells were collected and stored at -80°C for extraction of RNA.

### Metastasis definition

As previously mentioned, metastasis was confirmed by histopathology of the RLNs after different time points (n = 6 per time point). A group of six rabbits at a particular time point was considered “non-metastatic” if no metastasis was observed in all six animals. A group was considered to have “partial metastasis” if some animals had RLN metastasis while others did not. Finally, in those groups with “complete” metastasis, all six rabbits had RLN metastasis.

### Real-time quantitative RT-PCR

RNA was extracted from 50-100 mg of the tissue samples and 100 μg of the white blood cells using Trizol. cDNA was obtained using the RNA samples (2 μg) and a RT-PCR kit (Promega Corporation, Madison, WI) following the manufacturer’s instructions, which produced a reaction mixture of 20 μL. The conditions for reverse transcription were 70°C for 5 min and 42°C for 60 min. The diluted cDNA (2 μL) was then used for real-time PCR using the Platinum SYBR green q-PCR Super Mix–UDG (Invitrogen, Carlsbad, CA) along with the following primers, which were designed with Premier 5.0 and synthesized by the Shanghai Yingwei Jieji Co., Ltd (Shanghai, China): VEGF-c: 5′ CCCCAAACCAGTAACAATCAGT 3′ (forward), 5′ CTGGCAGGGAGCGTCTAAT 3′ (reverse); and GAPDH: 5′ AGAGCACCAGAGGAGGACG 3′ (forward), 5′ TGGGATGGAAACTGTGAAGAG 3′ (reverse). The conditions for the fluorescence real-time quantitative PCR were as follows: 95°C for 2 min; 45 cycles of 95°C for 30 s, 58°C for 30 s and 72°C for 30 s; and 95°C for 1 min, 58°C for 30 s and 95°C for 30 s. The relative expression of VEGF-c was calculated as follows: ΔCt (target gene) = Ct (target gene) – Ct (GAPDH). ΔΔCt=ΔCt (target gene) - ΔCt (standard) mean of target gene. The relative copies of the target gene were determined as 2^-ΔΔCt^.

### Statistical analyses

Continuous variables among more than two groups were compared by one-way analysis of variance (ANOVA). When a significant difference between groups was apparent, multiple comparisons of means were performed using the Bonferroni procedure with type-I error adjustment. Differences in VEGF-c mRNA expression between non-metastasized and metastasized RLNs were determined using an independent two sample t test. Data are presented as means ± standard deviation (SD). All statistical assessments were two-sided and evaluated at the 0.05 level of significant difference. Statistical analyses were performed using SPSS 15.0 statistics software (SPSS Inc, Chicago, IL).

## Results

### Establishment of an animal model of EC with RLN metastasis

**Figure 1:**
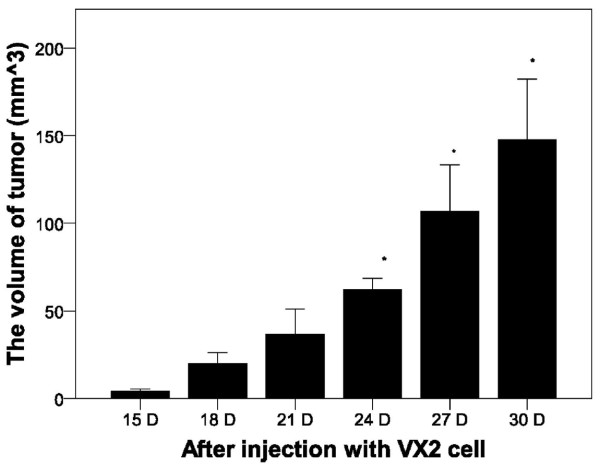
**Tumor volume over time after injection with VX2 tumor cells.** The size of tumors in the experimental group was determined at the indicated time points. Results represent the means ± SD at each time point (n = 6 per time point). ^*^ Indicates a statistically significant difference between the indicated group and the 15 D group, *P* < 0.05.

**Figure 2:**
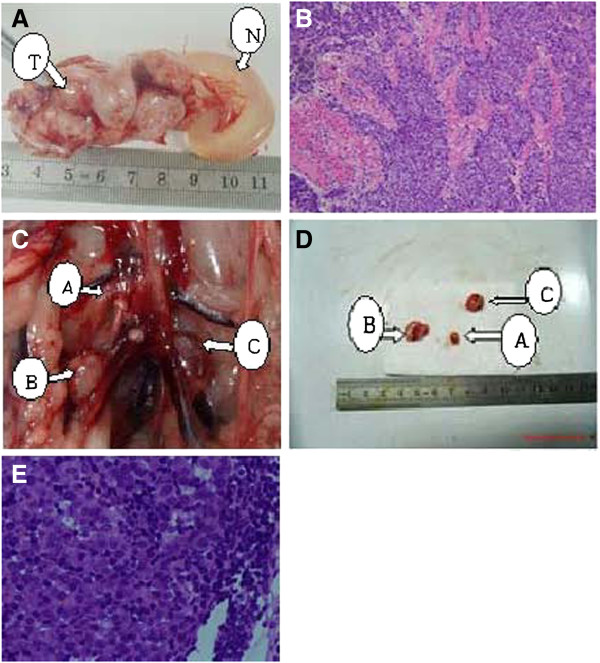
**Macroscopic and pathological analysis of rabbit endometrium and RLNs. A**: At day 21, a tumor was observed within the endometrium. No other tumors were observed within the surrounding tissues. T: endometrial tumor; N: normal uterine body. **B**: Histological examination of the endometrium at day 21 revealed orthotopic EC (H&E, 200×). **C**: 21 days of retroperitoneal lymph nodes metastasis. Arrow A shows the non metastasic RLN. Arrows B &C show the pathological confirmed metastasic RLN. **D**: After isolations of RLNs, the non-metastatic RLN had no increased volume as the metastatic RLN. **E**: Histological examination day 21 group rabbit model of peritoneal lymph nodes (microscopic, HE, 400x).

As shown in Figure 1, significantly increased tumor volume was observed at days 24, 27, and 30 post-injection of VX2 cells (*P* < 0.05). A representative image of the normal and tumor endometrium after 21 days is shown in Figure 2A. Histological analysis of the tumor tissue confirmed the presence of tumor cells (Figure 2B).

The presence of RLN metastasis was also assessed. RLN enlargement was not observed macroscopically at 15 days post-VX2 cell injection; however, pathological examination confirmed the absence of RLN metastasis (Table 1). At days 18 and 21 post-VX2 cell injection, enlargement of several RLNs was observed (Figure 2C); however, pathological examination revealed the absence of RLN metastasis at the 18-day time point (Table 1). The presence of non-metastasis RLN and metastasis into several RLNs was observed at 21 days post-injection (Figure 2C, 2D; Table 1). Enlargement of all RLNs and metastasis was noted at days 24, 27 and 30 post-injection (Table 1).

**Table 1:** Development of RLN metastasis over time in an in vivo model of orthotopic endometrial cancer

	**The frequency of metastasized/examined lymph nodes (n = 6 per time point)**					
	**No. 1**	**No. 2**	**No. 3**	**No. 4**	**No. 5**	**No. 6**
*Post-injection*						
*15 D*	0/1	0/1	0/2	0/1	0/1	0/2
*18 D*	0/3	0/3	0/2	0/1	0/1	0/2
*21 D*	1/3	0/3	0/3	2/3	0/3	1/3
*24 D*	3/3	3/3	3/3	3/3	3/3	3/3
*27 D*	3/3	3/3	3/3	3/3	3/3	3/3
*30 D*	3/3	3/3	3/3	3/3	3/3	3/3

### VEGF-c expression in the tumor, retroperitoneal lymph node, and peripheral white blood cells

**Figure 3:**
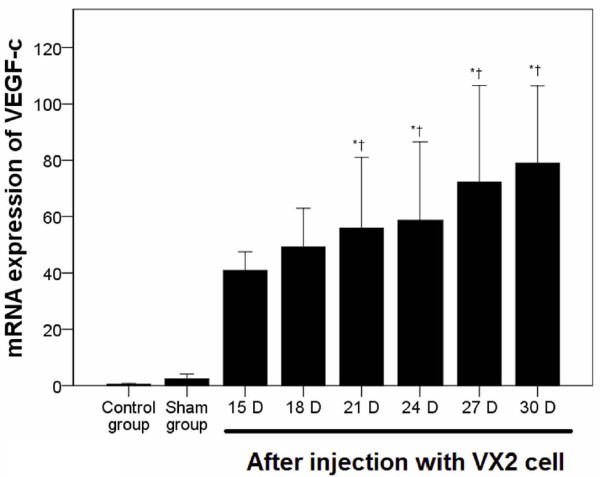
**VEGF-c mRNA expression in a rabbit endometrial tissue and in EC.** VEGF-c mRNA expression in the original tumor site was determined using quantitative RT-PCR over time. Pair-wise multiple comparisons between groups were determined using Bonferroni’s test with α = 0.001 adjustment. Results represent the means ± SD at each time point (n = 6 per time point). Indicates a statistically significant difference between the indicated group and the ^*^normal control group and ^†^ saline group.

VEGF-c mRNA expression was determined by quantitative RT-PCR. As shown in Figure 3, no significant difference in VEGF-c expression in the endometrial tissue was observed between the normal control and saline groups. As compared with the normal control and saline groups, VEGF-c mRNA expression in the EC tissue was markedly increased at 21 days post-injection (*P* < 0.001, Figure 3).

**Figure 4:**
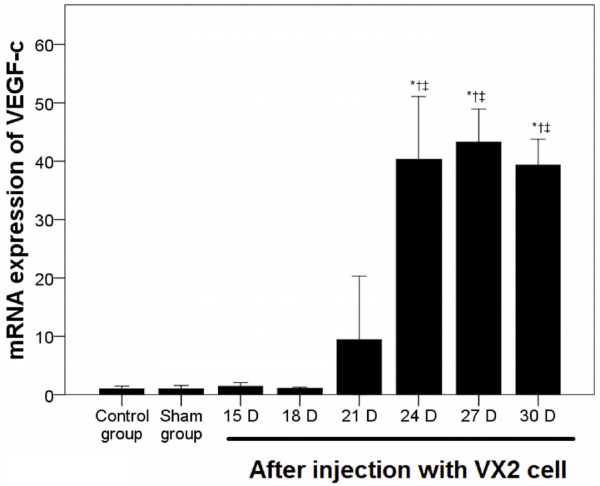
**Increased VEGF-C mRNA levels in retroperitoneal lymph nodes.** VEGF-c mRNA expression in the retroperitoneal lymph nodes was determined using quantitative RT-PCR over time. Pair-wise multiple comparisons between groups were determined using Bonferroni’s test with α = 0.001 adjustment. Results represent the means ± SD at each time point (n = 6 per time point). Indicates a statistically significant difference between the indicated group and the ^*^normal control group, ^†^ saline group, ^‡^ VX2 cell group at 15 days.

**Figure 5:**
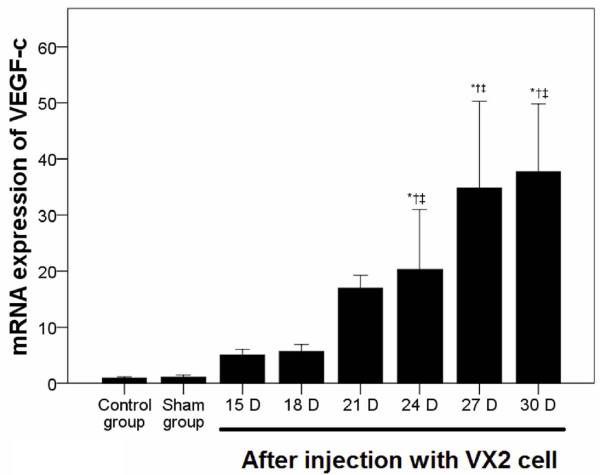
**VEGF mRNA expression in peripheral white blood cells.** VEGF-c mRNA expression in the peripheral white blood cells was determined using quantitative RT-PCR over time. Pair-wise multiple comparisons between groups were determined using Bonferroni’s test with α = 0.001 adjustment. Results represent the means ± SD at each time point (n = 6 per time point). Indicates a statistically significant difference between the indicated group and the ^*^normal control group, ^†^ saline group, ^‡^ VX2 cell group at 15 days.

**Figure 6:**
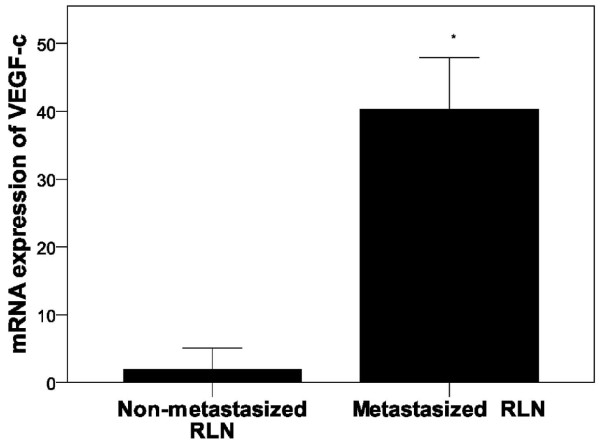
**mRNA expression of VEGF-c in non-metastatic and metastasized RLNs.** VEGF-c mRNA expression was determined using quantitative RT-PCR in non-metastatic (n = 29) and metastasized RLNs (n = 19). Results represent the means ± SD. ^*^ Indicates a statistically significant difference between the non-metastatic and metastasized RLNs, *P* < 0.05.

In RLN tissues (Figure 4) as well as peripheral white blood cells (Figure 5), again no marked difference in VEGF-c mRNA expression was observed between the normal control and saline groups. However, a dramatic increase in VEGF-c mRNA expression in RLN tissues was observed 24 days post-injection (*P* < 0.001, Figure 4). As compared to non-metastatic RLN tissue, significantly higher VEGF-c mRNA expression was observed in RLNs with metastasis (*P* < 0.001, Figure 6).

**Figure 7:**
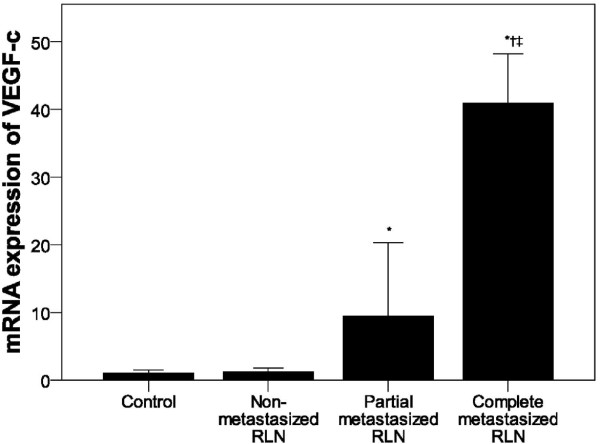
**VEGF-c mRNA expression in peripheral white blood cells by RLN status.** VEGF-c mRNA expression in the control (n = 12), non-metastatic (n = 12) and partial metastasized RLNs (n = 6), and complete metastasized (n = 18) groups was determined using quantitative RT-PCR. Pair-wise multiple comparisons between groups were determined using Bonferroni’s test with α = 0.008 adjustment. Results represent the means ± SD. Indicates a statistically significant difference between the indicated group and the ^*^control group or ^†^ non-metastatic RLN group, *P* < 0.05. ^‡^ Indicates a statistically significant difference between the partial and complete metastatic RLN groups, *P* < 0.05.

A similar increase in VEGF-c mRNA expression was noted in peripheral white blood cells at 24 days post-injection as compared to the control groups (*P* < 0.001, Figure 5). In addition, significantly higher VEGF-c mRNA expression was observed in peripheral white blood cells of rabbits with metastasis to all RLNs as compared to those without RLN metastasis (*P* < 0.002, Figure 7). A difference in VEGF-c mRNA expression was also observed between groups with partial and complete RLN metastasis (*P* < 0.008, Figure 7).

## Discussion

Because RLN metastasis is an important factor in determining the prognosis of EC [2-4], the relationship between VEGF-c mRNA expression and RLN metastasis was evaluated in an in vivo model in the present study. The establishment of EC with RLN metastasis was confirmed at day 21 post-injection with VX2 cells. In addition, VEGF-c mRNA expression increased significantly over time in the tumor site, RLN tissue, and peripheral white blood cells. Metastatic RLNs expressed higher VEGF-c mRNA expression as compared to those without metastasis. Furthermore, significantly higher VEGF-c mRNA expression was observed in peripheral white blood cells of rabbits with RLN metastasis, indicating that VEGF-c levels may have predicative value for metastasis in EC.

The VX2 cell line is composed of squamous cell carcinoma cells derived from Shope virus-induced papilloma in rabbit [13]. Their high survival rates make them a suitable candidate for in vivo inoculation [13], which has been carried out in liver, lung, uterus, and breast tissues to establish the corresponding animal models [14-16]. In the endometrium, inoculation of VX2 cells induced EC with lymph node metastasis after 14-21 days post-injection [14,15], which is similar to the results of the present study. These previous studies employed blocks of VX2 cells in the myometrium using microsurgical instruments while the present study introduced the VX2 cells via an injection, which removes the dependence on microsurgical instruments, is less difficult, and may increase the success rate of establishing the model as was evident in the 100% success rate of establishing EC and RLN metastasis observed in this study. The high success rate of this study using this method is consistent with results reported by Chen et al. [16].

In addition to the advantage of a high success rate, the in vivo model of EC employed in the present study induced EC with lymph node metastasis by day 21, which was faster than that reported for other models. For example, establishment of a mouse model of EC with lymph node metastasis via 5 million HEC1A cells required eight weeks [8]. Of note, 100% of the animals in the present study developed EC with RLN metastasis, which was greater than that observed using the HEC1A mouse model of 86.5% [8].

In the present study, an in vivo model of EC with RLN metastasis was established by injecting 500 million VX2 cells into the uterine muscularis mucosae of rabbits. Previous studies in our laboratory indicated that this number of cells was necessary for successful RLN metastasis (data not shown). Analysis of the peritoneal cavity revealed that primary tumors were restricted to within the uterus as peritoneal dissemination of the VX2 cells was not observed.

Tumor angiogenesis is related to its invasion and metastasis [17], and VEGF is an important factor regulating angiogenesis in cancers [18]. The *VEGF-c* gene is mapped to chromosome 4q34 [19], and in vitro studies have confirmed that it induces lymphangiogenesis but not angiogenesis [20]. Straume et al. [21] also reported that lymphatic vessel density in nodular melanoma was positively related to the VEGF-c level. Furthermore, previous studies have shown that the VEGF-c expression in solid cancers, including endometrial adenocarcinoma, was significantly higher than that observed in adjacent normal tissues [22-24], which is consistent with the data presented in this study. In patients with gastro-oesophageal junction adenocarcinoma, VEGF-c levels were associated with tumor stage, lymph node metastasis, and shorter periods of disease-free survival [25]. Further studies will assess if the increase in VEGF-c expression is directly related to EC growth and metastasis through lymphangiogenesis. In addition, a possible synergism between VEGF-c and FGF-2 as reported by Cao et al. [26] will be explored.

In the present study, differences in VEGF-c mRNA expression were observed among rabbits without and with RLN metastasis; differences were also noted between rabbits with full and partial metastasis with the highest expression found in rabbits with metastasis to all RLNs. These results suggest that the increased VEGF-c may arise from the EC during its development and progression, which may subsequently promote lymph node metastasis [27]. This data is consistent with that reported by Kimura et al. [10] in which VEGF-c levels correlated with lymph node metastasis and lymphatic involvement in esophageal squamous cell carcinoma. Although VEGF-c expression increased with tumor growth and RLN metastasis, further studies will evaluate whether its level is reflective of the cancer load and metastatic state of EC.

The current study was limited in that it did not confirm whether VEGF-c protein levels were correlated with those obtained for mRNA. Further studies will evaluate the VEGF-c protein levels, as well as markers of epithelial-mesenchymal transition (EMT), in tissues as well as serum samples using immunohistochemistry and enzyme-linked immunosorbent assays (ELISAs). In addition, although previous studies have reported a relationship between VEGF-c and lymphangiogenesis with metastasis [6-8], no mechanistic data were presented in this study. Finally, VEGF receptor expression was not determined.

## Conclusions

Thus, an animal model of EC with RLN metastasis was established, which revealed an increase in VEGF-c mRNA expression in peripheral white blood cells with tumor growth and metastasis. Should further studies determine that VEGF-c levels are reflective of tumor load and metastatic state, examining serum VEGF-c levels may be useful for predicting RLN metastasis in EC patients and further inform clinicians as to the most effective treatment option, including intra-operative RLN dissection.

## Abbreviations

ANOVA: Analysis of variance; EC: Endometrial cancer; ELISAs: Enzyme-linked immunosorbent assays; RT-PCR: Real-time quantitative PCR; RLN: Retroperitoneal lymph node; SD: Standard deviation; VEGF-c: Vascular endothelial growth factor c; VEGFR-3: VEGF receptor-3.

## Competing interests

The authors declare that they have no competing interests.

## Authors’ contributions

YWH: study concepts; study design; definition of intellectual content; literature research; clinical studies; experimental studies; data acquisition; data analysis; statistical analysis; manuscript editing. LQX: study concepts; literature research; experimental studies; data acquisition; data analysis; statistical analysis; manuscript preparation. RZL: experimental studies. XH: clinical studies. TH: experimental studies. YNZ: guarantor of integrity of the entire study; study concepts; study design; definition of intellectual content; clinical studies; manuscript review. All authors read and approved the final manuscript.
